# Improving the Quality of Wheat Flour Bread by a Thermophilic Xylanase with Ultra Activity and Stability Reconstructed by Ancestral Sequence and Computational-Aided Analysis

**DOI:** 10.3390/molecules29081895

**Published:** 2024-04-22

**Authors:** Guoshuai Hu, Xizhi Hong, Meixin Zhu, Lei Lei, Zhenggang Han, Yong Meng, Jiangke Yang

**Affiliations:** 1 Pilot Base of Food Microbial Resources Utilization of Hubei Province, College of Life Science and Technology, Wuhan Polytechnic University, Wuhan 430023, China; hudou233@163.com (G.H.); zmx18062521684@163.com (M.Z.);; 2 Mianyang Habio Bioengineering Co., Ltd., Mianyang 621000, China; mengyong@habio.net

**Keywords:** thermophilic xylanase, ancestral sequence reconstruction, bread texture improved, yeast fermentation

## Abstract

Xylanase is an essential component used to hydrolyze the xylan in wheat flour to enhance the quality of bread. Presently, cold-activated xylanase is popularly utilized to aid in the development of dough. In this study, ancestral sequence reconstruction and molecular docking of xylanase and wheat xylan were used to enhance the activity and stability of a thermophilic xylanase. The results indicated that the ancestral enzyme TmxN3 exhibited significantly improved activity and thermal stability. The Vmax increased by 2.7 times, and the catalytic efficiency (K_cat_/K_m_) increased by 1.7 times in comparison to TmxB. After being incubated at 100 °C for 120 min, it still retained 87.3% of its activity, and the half-life in 100 °C was 330 min, while the wild type xylanase was only 55 min. This resulted in an improved shelf life of bread, while adding TmxN3 considerably enhanced its quality with excellent volume and reduced hardness, chewiness, and gumminess. The results showed that the hardness was reduced by 55.2%, the chewiness was reduced by 40.11%, and the gumminess was reduced by 53.52%. To facilitate its industrial application, we further optimized the production conditions in a 5L bioreactor, and the xylanase activity reached 1.52 × 10^6^ U/mL culture.

## 1. Introduction

Xylan is a type of heteroglycan that contains a chain of 1,4-β-d-xylopyranosyl units as a backbone, along with side-chain groups like arabinose and glucuronic acid [1]. It makes up around 3% of wheat flour. However, the insoluble xylan can compete with gluten for water, which can interfere with the formation and development of the gluten network, ultimately being detrimental to the bread structure [2].

The biodegradation of xylan involves several xylanolytic enzymes [3], including endo-β-1,4xylanase (EC 3.2.1.8), 1,4-β-xylosidase (EC 3.2.1.37), α-glucuronidase (EC 3.2.1.139), and α-l-arabinuronidase (EC 3.2.1.55) [4]. Among them, endo-1,4-β-xylanase plays a crucial role and stochastically cleaves the backbone of xylan into short xylo-oligosaccharides [5]. Xylanase has numerous benefits in the food industry [6]. When mixed with wheat flour, xylanase can hydrolyze the glycosidic chain of wheat xylan to make it water-soluble. This can reduce the water holding capacity of non-starch polysaccharides in wheat flour, ultimately improving the performance of refined wheat bread [7]. Moreover, oligosaccharides produced by xylanase may also add potential health benefits to bread. Therefore, xylanase has become an indispensable component in wheat flour additives to improve the quality of flour products [8].

Cold-activated xylanases are currently more popular in wheat flour as dough development usually occurs at low temperatures. However, this reason seems to overlook the crucial importance of the later high-temperature baking process, which might also be important for the development of bread [9]. Although the thermal resilience of the biocatalyst confers various benefits, such as augmented reaction rates and enhanced substrate solubility [10,11], the xylanase has a higher optimal temperature; the thermophilic enzyme, especially, is less used in the betterment of wheat flour products [12]. It could be speculated that thermophilic xylanase with certain activity under room temperature could fully act on the xylan in the high-temperature baking process, which might also be used in the improvement of flour products [13]. Although many studies have been conducted on the impact of mesophilic xylanases on bread making, there has been limited research on thermostable xylanases. Current thermostable enzymes still lack sufficient thermal stability for high-temperature baking. Ancestral sequence reconstruction is a newly developed semi-rational method for modifying proteins. This method uses different models to create a phylogenetic tree and make inferences about the amino acids of ancestral protein nodes. Enzymes that are reconstructed based on the ancestral sequence usually exhibit better stability and can achieve 30–40 °C higher temperature stability than the original protein after modification, which makes them expected to exhibit extreme properties.

Ancestral sequence reconstruction has emerged as a useful methodology for engineering proteins with enhanced properties [14]. Previous studies on enzymes such as alcohol dehydrogenase KpADH [15], laccase AnCotA2 [16], and xylanase [17] have all recorded improved stability and a broader substrate spectrum. However, care should always be taken since reconstructed sequences represent historical proteins. The reconstruction can lead to non-functional, multifunctional, promiscuous, or nonviable sequences [14]. It remains unclear whether ancestral proteins are generally more promiscuous or multifunctional than modern proteins. Among the ancestors, the biased substrate sets and the specificity on the substrates should also be assayed [18,19]. Molecular dynamic analysis between enzymes and substrates allows protein engineers to mine functionally rich regions of sequence space for target substrates [20] and to identify protein sequences that confer a defined ligand specificity profile. Thus, unique or promiscuous activities like these can be optimized [21].

In this study, the ancestral sequences of TmxB, a thermophilic xylanase isolated from *Thermotoga maritima*, were redesigned. Molecular dynamic analysis was conducted between enzyme and substrate to improve its activity and stability under extreme thermal conditions. Its application in the betterment of wheat flour bread was evaluated. The production of the enzyme in a 5L bioreactor was also systematically conducted to facilitate its future industrial application.

## 2. Results

### 2.1. Inferring the Ancestral Sequences Based on the Evolutionary Branches and Molecular Dynamic Analysis

**Figure 1:**
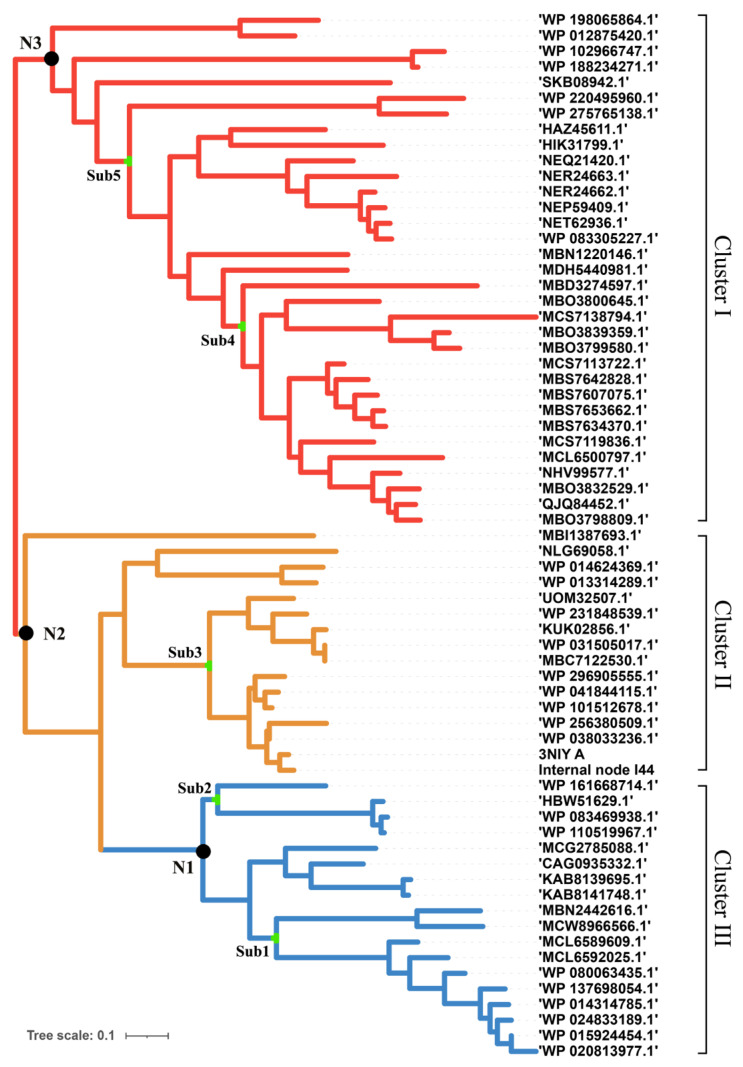
Phylogenetic tree of xylanase to construct the ancestral sequences. There are different branches according to sequence similarity. Three genes were selected for subsequent experiments.

*T. maritima* endo-1,4-beta-xylanase B and the reference sequences with homology greater than 40% were extracted from the protein database of the NCBI. The CD-hit was used to remove repetitive parts of the sequence. A maximum likelihood phylogenetic tree was constructed based on the distance matrix generated from the multi-alignment (Figure 1). According to the results, it is known that xylanase has broad branches and shows different evolutionary directions. These xylanases were grouped into three clusters, I, II, and III. Xylanases such as the enzyme (ID: MBS7653662) from the *Candidates Bathyarchaeota archaeon*, which was isolated from hot spring sediment, were ascribed to Cluster I. Enzyme (ID: 3NIY), characterized with thermal stability from *Thermotoga petrophila* RKU-1, and the related xylanases were ascribed to Cluster II. The xylanases such as those from *Ruminiclostridium cellulolyticum* (WP_015924454), *Herpetosiphon llansteffanensis* (WP_110519967), and related sequences, were ascribed to Cluster III.

**Figure 2:**
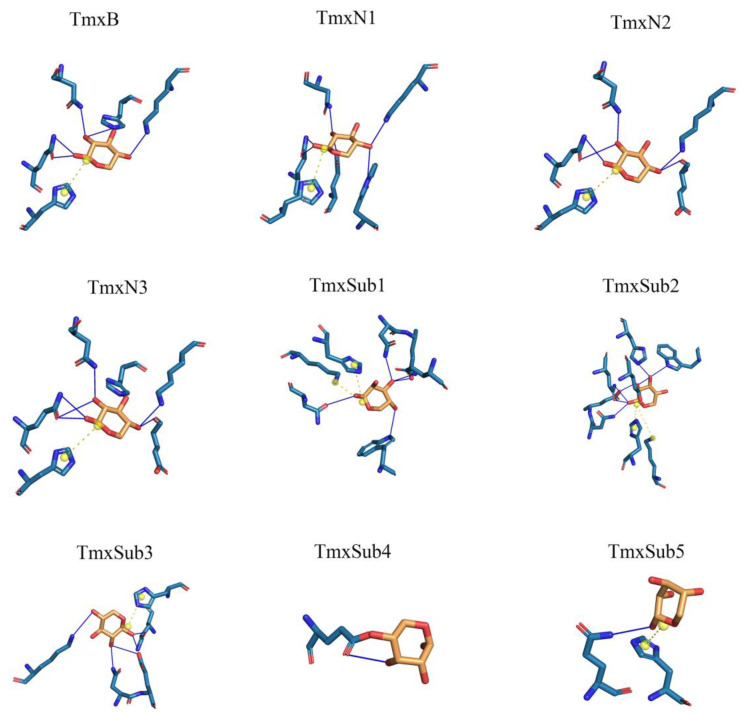
A thorough examination of the amino acid interactions taking place within the active site of both the wild type and ancestral enzymes. Specifically, we honed in on the two neighboring sugar substrate binding sites and the associated residues situated around the cleavage site. Our findings are visually represented through a diagram featuring solid blue lines for hydrogen bond interactions and gray dashed lines for hydrophobic interactions.

According to the phylogeny distance, eight ancestral sequences from the cluster nodes were selected for molecular dynamic analysis with xylan as the ligand (Figure 2, Table 1). The substrate xylan had the highest number of hydrogen bonds with TmxN3 (seven), while TmxB, TmxN1, and TmxN2 formed six, six, and five hydrogen bonds, respectively. The archaea-derived HBW and MBN also formed seven and five hydrogen bonds, while 3NIY from the thermophilic cluster formed five hydrogen bonds. Conversely, xylanases MBD and SKB only formed a single hydrogen bond. Salt bridges were formed in all xylanases except MBD. Xylan’s hydroxyl groups allow for hydrogen bonding with polar amino acids, increasing affinity. The archaea’s active pocket needs to ensure thermal stability while interacting with high-temperature xylanases, resulting in relatively more hydrophobic amino acids and lower affinity with xylan. Low-temperature xylanases have greater flexibility in their active pockets, leading to higher affinity. Evolution may have enhanced high-temperature xylanase affinity with xylan while maintaining thermostability. Mutants 1 and 3 showed higher affinity than the wild type after mutation. Therefore, TmxN1 was assumed as the ancestral sequence of Cluster III, TmxN2 was the ancestral sequence of Cluster II and III, and TmxN3 was the ancestral sequence of Cluster I, and was selected for experimental determination.

### 2.2. Ancestral Xylanases Exhibited Significant Improvement in Thermal Stability and Activity

**Figure 3:**
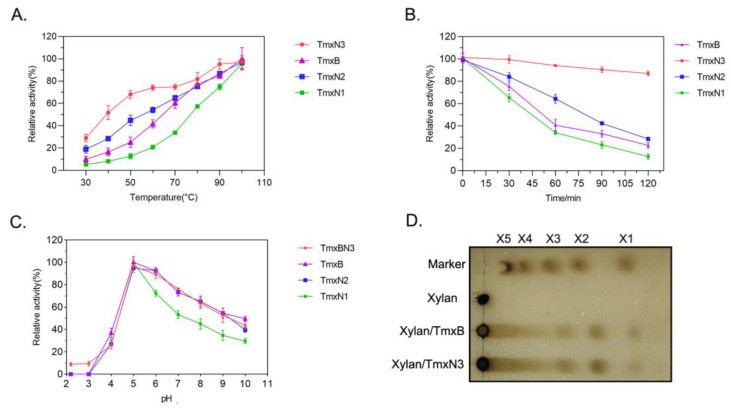
Enzymatic characteristics of xylanases. (**A**): Enzyme activity of xylanases under different temperatures. (**B**): The activity of xylanases at different time points after incubating at 100 °C (in boiling water). (**C**): Enzyme activity of xylanases under different pHs. (**D**): TLC analysis of the products of xylan digested by xylanases.

The enzymatic characteristics including the activity, thermal stability, and the kinetic parameters of xylanases were determined in this study (Figure 3, Table 2). The optimal temperature of all the enzymes, including TmxB and three ancestral xylanases, was found to be 100 °C (in boiling water), indicating their thermophilic nature. However, TmxN3 and TmxN2 exhibited higher activity than the TmxB enzyme at temperatures ranging from 30 to 90 °C, with their activity transition being smoother than that of TmxB. Conversely, TmxN1 had lower activity than TmxB (Figure 3A).

The stability of the ancestral sequence TmxN3 was found to be significantly better than TmxB. Even at a temperature of 100 °C (boiling water) and after being incubated for a period of 120 min, TmxN3 retained 87% of its enzymatic activity. In contrast, the wild type TmxB enzyme retained only 25% of its activity under similar conditions. TmxN2 showed slightly better thermal stability than TmxB, while TmxN1 showed weak stability (Figure 3B). The calculated half-life time of the xylanases further showed that the half-life of TmxN3 at 100 °C was 330 min, while the wild type xylanase was only 55 min. 

All the enzymes exhibited optimal activity at pH 5.0 (Figure 3C). After reacting for 10 min at 100 °C and pH 5, both xylanases TmxB and TmxN3 were found to hydrolyze wheat arabinoxylan into xylose, xylobiose, and xylotriose, with the hydrolysis products mainly being xylobiose and xylotriose, as determined by TLC (Figure 3D). 

The kinetic parameters of TmxB and the ancestral sequences for wheat xylan were tested at 100 °C and pH 5. Under these conditions, the maximum velocity (V_max_) of TmxN3 was found to be 13,958 U/mg, which was significantly higher than that of TmxB (17,019 U/mg) and TmxN2 (45,827 U/mg). TmxN3 increased the catalytic efficiency (K_cat_/K_m_) by 2.7-fold compared to TmxB (Table 2).

### 2.3. The Expression Level of Xylanases Improved by Increasing the Gene Dosage in Host Genome

**Figure 4:**
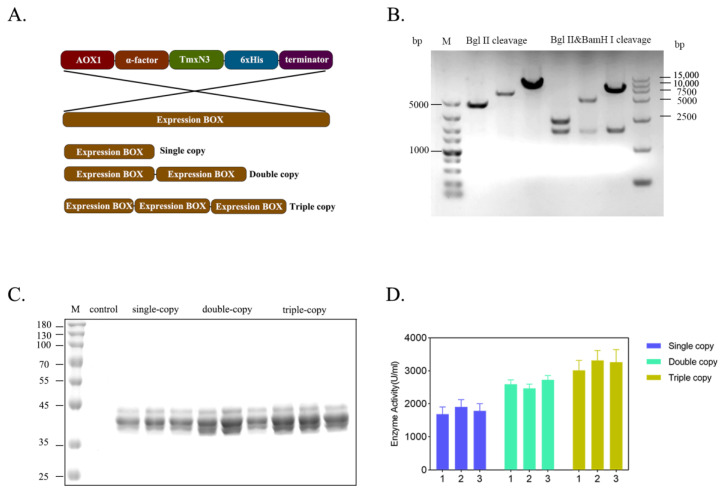
Improving the gene dosage of xylanase by constructing the concatenated expression cassettes of xylanase gene. (**A**): Organization of expression cassettes of xylanase gene. (**B**): Enzymatic check for the recombinants carrying the concatenated genes. (**C**): Protein profiles of xylanase expressed by the yeast recombinants in flask. (**D**): Enzyme activity of the recombinants expressed in flask.

To enhance the production of xylanases, we increased the gene dosage of xylase in the host genome by creating multiple copies of expression cassettes of xylanase (Figure 4A). We successfully screened and identified clones carrying single-, double-, and triple copies of xylanase genes through PCR amplification of the expression cassette, followed by enzyme digestion ligation (Figure 4B). Apparently, with the increase in the gene copies, the expression level of these recombinants was improved correspondently, and the enzyme activity of the single-copy was 1683.86 U/mL, the second-copy enzyme activity was 2598.63 U/mL, and the third-copy enzyme activity was 3263.84 U/mL, cultivated in flasks. This resulted in an overall increase in enzyme activity by 1.94 times (Figure 4C,D). However, the expression of protein secretion will be hindered as the copy number increases. Many incompletely folded proteins accumulate in *Pichia pastoris* or incompletely folded proteins are secreted out of the cell. Solving this problem involves deeper modifications to the chassis cells and more in-depth research.

### 2.4. Bioreactor Production of Thermalphilic Xylanase by High-Density Cultivation

**Figure 5:**
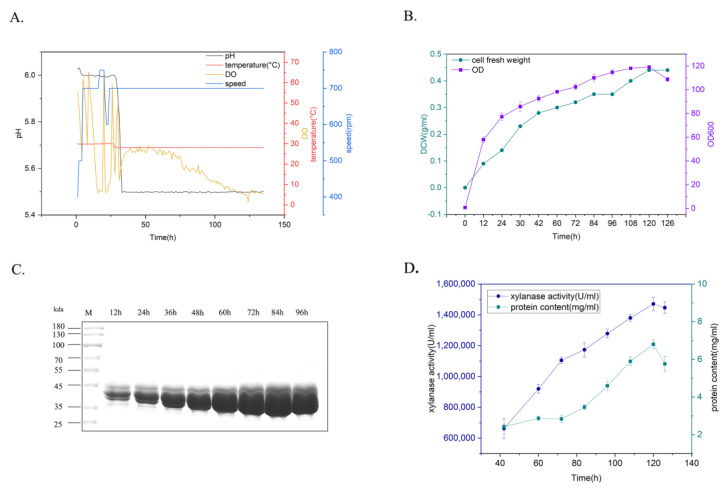
Expression of xylanase in 5L bioreactor. (**A**): Parameters for the cultivation of recombinants. (**B**): The time course of the changes of the cell fresh weight and the cell density (OD). (**C**): Protein profiles of the recombinant after inducible expression in the culture. (**D**): The time course of the changes of the xylanase activity and protein content in the culture.

To determine the production potential of xylanase for various industrial applications, yeast recombinants carrying xylanase TmXN3 were cultivated using a fed-batch process in a 5L bioreactor (Figure 5). The yeast recombinants were cultured mainly under the conditions of Tm = 28 °C and pH 5–5.5, and the agitation speed and aeration were coupled with dissolved oxygen. This process could be roughly divided into a glycerol feed stage and an inducible expression stage. After the glycerol was fully consumed, the dissolved oxygen (DO) levels rebounded. At this point, methanol was added into the culture for induction expression of xylanase, and samples were checked every 12 h (Figure 5A). After 126 h cultivation, the OD of the culture (cell density) reached 119, and the cell fresh weight reached 0.44 g/L (Figure 5B). The expression level gradually increased with the prolongation of time. The protein content and enzyme activity in the supernatant reached 6.8 mg/mL and 1,519,682 U/mL at 120 h (84 h induction), respectively, which was the highest xylanase activity achieved in shake flask fermentation (3263 U/mL) (Figure 5C,D). 

### 2.5. Thermophilic Xylanases Significantly Improved the Texture of Bread

**Figure 6:**
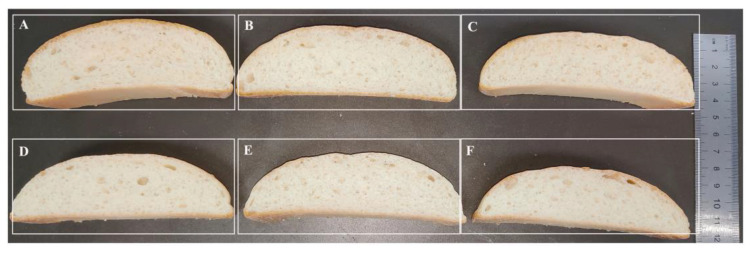
Phenotypes of bread treated with xylanases. (**A**–**C**): Xylanase TmxN3 was added with 4.5, 3.0, and 1.5 mg/kg of flour, respectively. (**D**,**E**): Xylanases of TmxB were added with 4.5 mg/kg and 1.5 mg/kg of flour, respectively. (**F**): Bread without added xylanase.

The effect of the thermophilic xylanases on the quality of wheat flour bread was systematically evaluated in this study (Figure 6, Table 3). Apparently, both the wild type and ancestral xylanases were found to significantly improve the texture of bread, and apparently the bread treated by xylanase TmxN3 had the height of 4.5 cm, while the height of the control bread was 3.5 cm. The addition of xylanases TmxN3 reduced the bread’s hardness from 508.28 g to 228.64 g, the chewiness from 370.07 g/mm to 165.99 g/mm, and the gumminess from 418.69 to 189.08. However, the springiness, cohesiveness, and resilience of the bread were not significantly changed with the addition of xylanase.

These breads were further stored on the shelf until to 10 d and the effects of xylanase on the textural properties of bread were also investigated (Table 3). Although the bread showed aging behavior, increased hardness and chewiness, and decreased elasticity, resilience, and adhesion during 1, 7, and 10 d of storage, the xylanase TmXN3-treated bread showed better performance when stored on the shelf than the untreated or TmxB-treated bread. After 10 d, the bread treated by 4.5 mg/kg xylanase had better hardest, chewiness, and gumminess than the controls.

## 3. Discussion

### 3.1. Ancestral Sequences Reconstruction and Molecular Dynamics Analysis Improved the Activity and Thermostability of Xylanase

Ancestral sequence reconstruction is a popular method used to redesign and modify proteins. Since the ancient Earth was an extremely hot environment, ancestral proteins often display desirable characteristics, such as enhanced thermostability and activity. By constructing a phylogenetic tree and inferring the amino acids of ancestral protein nodes, it is possible to find more stable proteins among these ancestral sequences. Reconstructed ancestral proteins, like the xylanase AncXyl09, have been shown to have improved stability, with a half-life of 65.08 h at 50 °C. Similarly, the designed AncCotA2 based on the ancestral sequence showed higher thermal and acid stability [16]. Moreover, ancestral enzymes may have a broader substrate spectrum than the original enzymes.

However, not all ancestral sequences are better than their modern counterparts. Enzyme XynN1, for instance, the ancestral sequence of Cluster III, did not exhibit better thermostability. A deep analysis of this cluster found that xylanases isolated from strains such as *Ruminiclostridium cellulolyticum* and *Herpetosiphon llansteffanensis* usually had normal temperature preferences. On the other hand, enzyme TmxN2, the ancestral sequence of Cluster II, contains xylanases such as enzyme (ID: 3NIY) from *Thermotoga petrophila* RKU-1, which originated from thermal environments. The ancestral sequence TmxN3 was assumed to be the ancestral sequence for xylanase such as ID: MBS7653662 from the Candidates Bathyarchaeota archaeon, which exhibited excellent thermostability. As tested in this study, TmxN3 exhibited ultra thermostability, and retained 87.3% of its activity after incubation at 100 °C for 2 h, while TmxB retained only 23% of its original activity. This demonstrates that ancestral sequence reconstruction is effective in improving the high-temperature resistance properties of enzymes.

**Figure 7:**
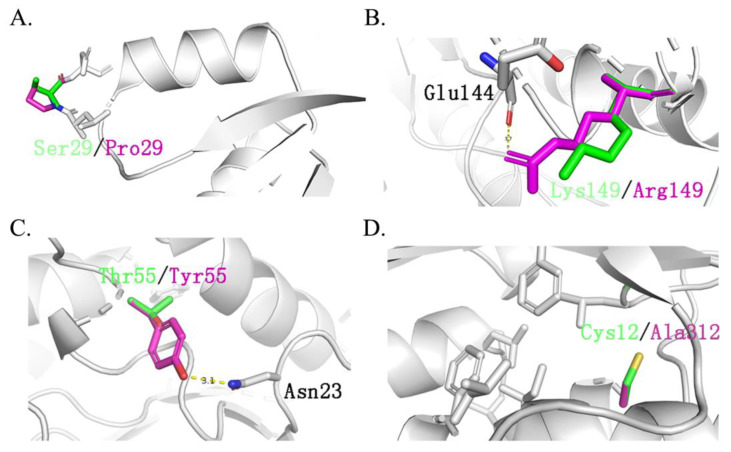
Main different mutation sites of TmxBN3 and TmxB. Green represents the original amino acid, while purple represents the mutated amino acid. (**A**): Ser29 located between β1-α1 is replaced by Pro29. (**B**): Asp139 replaced Ser139 and formed a hydrogen bond with Glu144. (**C**): Tyr55 replaced Thr55 and formed a hydrogen bond with Asn29, Asp139 replaced Ser139 and formed a hydrogen bond with Glu144, which also improved the stability of the structure. (**D**): Cys312 is replaced by Ala312, forming a hydrophobic core with Leu302, Phe280, Leu6, Lle316, and Phe266, which greatly improves the stability of the protein structure.

It should be noted that ancestral sequences might contain non-functional, multifunctional, promiscuous, or nonviable sequences. This is because reconstructed sequences represent historical proteins with functional divergences. Therefore, these ancestral sequences should be optimized further to reduce the possible promiscuous or multifunctional sequences. Thus, to overcome these drawbacks, in this work, nine ancestral sequences were initially reconstructed and screened through dynamic analysis among the enzyme and substrate. This step efficiently narrowed the candidates’ sequences down to three. The results of molecular docking showed that both mutants TmxN3 and TmxN3 had cross-linking of Glu48 and Trp281 to the substrate compared to TmxB, as well as the formation of more hydrogen-bonded networks. Meanwhile, the 85His to donor distance was 2.79 Å in mutant TmxN3, 2.80 Å in mutant TmxN3, and 3.98 Å in wild type TmxB. This may account for the increased Kcat/K_m_. It is noteworthy that we mutated Ser29 to Pro29, which greatly enhanced the stability of the protein (Figure 7). Further experiments showed the improvement of the activity and kinematics parameters, which further testified to the success of our strategies.

### 3.2. Xylanase TmxN3 Exhibited Excellent Thermal Stability

Xylanase, especially endo-1,4-β-xylanase, has several benefits in the food industry. By breaking down xylan, xylanases can reduce the viscosity of plant components, increase reducing sugars, and provide good antioxidant properties to the formulas [6]. Therefore, it can be used for bread making, saccharification of the mash, non-starch polysaccharide degradation, juice clarification, and other applications [22]. Although thermophilic enzymes are less commonly used to improve wheat flour products [12], thermophilic xylanase has great potential in many industrial applications beyond flour improvement, such as in feed, bio-bleaching, textiles, etc., which require high temperatures for the process [23]. Hence, high-temperature-resistant xylanase has significant production potential and application prospects [24]. 

Presently, a list of thermostable xylanases has been produced by a number of thermophilic (optimum temperature ranges 50–80 °C) and hyperthermophilic organisms (optimum temperature more than 80 °C), such as *Bacillus stearothermophilus*, *Thermoascus aurantiacus*, *Thermotoga* sp., and *Thermomyces lanuginosus*, etc. But these xylanases resources generally had optimal temperatures of 85 and 80 °C, and less than 100 °C. Also, one of the most thermostable GH-10 xylanases has been reported from *Thermotoga* sp. strain FjSS3-B.1 with an optimum temperature of 105 °C and a half-life of 90 min at 95 °C [6]. In contrast, the reconstructed ancestral sequence TmXN3 has ultra thermostability. As the temperature increased, the activity of TmxN3 steadily increased under pH 5 conditions, reaching maximum enzyme activity at 100 °C. The graph illustrates that TmxN3 maintained its stability even when subjected to an environment of pH 5 and 100 °C. The enzyme’s activity remained constant even after half an hour of incubation. After 60 min, there was only a 5% reduction in the enzyme’s activity. With continuous incubation for 120 min, TmxN3 still retained 87% of its activity. And the half-life of TmxN3 was 330 min at a pH 5 of 100 °C. This increased thermostability of xylanase is probably due to modification in its protein structure such as an increased number of hydrogen bonds and salt bridges, an increased number of charged surface residues, and an improvement in internal packing (Table 1, Figure 7). Moreover, the highest temperature checked in our study was 100 °C, because this was the highest temperature that our equipment could supply for the liquid enzyme. We believe that the ultra thermostable TmxN3 might have an optimal temperature higher than 100 °C.

To facilitate the future industrial application of xylanase TmXN3, we finished it with a 5L bioreactor cultivation and expression. Similar work conducted on a *Bacillus pumilus* alkaline stable xylanase in a 5L fermentor reached a 48,241 U/mL expression level [25]. Some other works on thermotolerant xylanases from *Aspergillus* sp. BCC125 [26], *Aspergillus sulphureus* [27], *Ruminococcaceae*, *Prevotella*, and *Lactobacillu* [28] have also reached a relatively high expression level. In our study, we successfully achieved the expression of thermophilic xylanase TmxN3 with enzyme activity that reached the ultra 1.52 × 10^6^ U/mL at 100 °C, and the protein content of 6.80 mg/mL in a 5L bioreactor. The protein in *P. pastoris* relies heavily on the addition of methanol, which is seriously toxic to the human body. The complete removal of methanol in downstream production is a very big problem, and its retention in proteins will limit its application in the food industry.

### 3.3. The Thermophilic Xylanases Effectively Improve Wheat Bread Quality

In general, wheat flour contains about 3% xylan, which is insoluble and can compete with gluten for water. This interferes with the formation and development of dough and can be detrimental to the structure of baked bread [29]. Xylanase, especially endo-1,4-β-xylanase, can hydrolyze the glycosidic chain of wheat xylan, making it water-soluble. This reduces the water holding capacity of non-starch polysaccharides in wheat flour and improves the performance of refined wheat bread [8] (Table 4). Additionally, xylanase releases xylo-oligosaccharides from flour, which have pre-biotic effects and stimulate the growth of intestinal bifidobacterial, promoting intestinal health [6].

The bread preparation process usually involves three stages: mixing, dough development (fermentation), and baking. Cold-activated xylanases are currently more popular for dough development, which typically occurs at 30–40 °C. However, during the baking stage with temperatures ranging from 150 °C to 210 °C, the enzyme is often deactivated. This overlooks the crucial importance of the high-temperature baking stage, during which the dough development continues. Thus, thermophilic xylanase, which still exhibits certain activity at 30–40 °C, could fully exert its function in the baking stage. Therefore, it could be speculated that thermophilic xylanase, which has certain activity under room temperature and acts on xylan in the high-temperature baking process, can also be used to improve flour products.

The results of the texture profile analysis are presented in Table 3. The findings indicate that with the addition of xylanase, the hardness, chewiness, and gumminess of bread reduced significantly in a dose-dependent manner. However, there was no impact on the cohesiveness, springiness, and resilience. The texture analysis of the bread samples revealed that the lowest hardness, chewiness, and gumminess were observed when the enzyme dosage was 4.5 mg/kg flour. It is worth noting that the recommended dosage of TmxN3 in bread baking is 4.5 mg/kg flour. Furthermore, the impact of xylanase on the texture of bread samples during storage at 4 °C was also studied, which is shown in Table 3. During the storage period of 1, 7, and 10 days, all bread samples showed aging behavior, with increased hardness and chewiness, and decreased elasticity and resilience. However, adding 4.5 mg/kg TmxN3 had a positive impact on the texture and quality of bread during the 10-day storage period. It significantly reduced the hardness and chewiness of bread. These results clearly demonstrate the positive impact of TmxN3 on bread properties. The structural properties of xylanase are closely tied to factors such as substrate specificity, inhibitor sensitivity, and temperature activity [12]. TmxN3 is a promising candidate for improving the texture of baked goods due to its high activity levels and ability to withstand high temperatures. Research has found that GH10 xylanase has a positive effect on bread volume at lower dosages and when combined with the textural properties of bread made using TmxN3, indicating that thermophilic xylanase has a selective advantage.

## 4. Materials and Methods

### 4.1. Constructing a Phylogenetic Tree of Xylanases and Ancestral Sequences Mining

The *T. maritima* xylanase B sequences (MT032174.1) and reference xylanases had a homology greater than 40% and coverage greater than 95%, and were obtained from the NCBI database. To eliminate redundant portions of the sequence, we employed CD-hit (version 4.8.1) with a threshold of 0.9 [33,34]. AliView software (Vesion 1.28) was used for multi-alignment [35], and 10,000 iterations were performed to create the phylogeny tree. Using the FastML (version 3.11) with a high posterior probability [36,37], we determined the most likely sequences from the same branch, and the ancestral sequences were obtained from the nodes as described before [16].

### 4.2. Molecular Dynamics Analysis of Xylanase and the Ligand

Three-dimensional models of wild type xylanase TmxB and its ancestral sequences were generated using Swiss-Model server (https://swissmodel.expasy.org/, accessed on 19 October 2023). According to the above analysis, xylanases with ID: 3NIY, HBW, MBN, MBD, and SKB were selected for the following analysis. Dynamic analysis of TmxB was performed using the crystal structure of TmxB (PDB entry: 3NIY_A, with water and ligand removed) as the receptor, and the corresponding protein models. The ligand was wheat xylan (https://pubchem.ncbi.nlm.nih.gov/, accessed on 19 October 2023). Wheat xylan molecules were docked to the gap in the active site of xylanase using AutoDock Tools 1.5.6 [38]. The docking grid covered glycone subsites from −1 to −4. Docking complexes were chosen based on binding energy for structural analysis. Intramolecular interactions of xylanase and its ancestral sequences were analyzed using PILP [39] server (http://plip-tool.biotec.tu-dresden.de/, accessed on 25 October 2023). The criterion for assigning hydrogen bonds was that the distance between the donor and acceptor atoms was less than 4 Å. All structural diagrams were produced using PyMOL (Version 2.5.0.) (Schrödinger).

### 4.3. Recombinants Construction and Expression of the Xylanases

The xylanase gene from *T. maritima* and its ancestral sequences, TmxN3, TmxN2, and TmxN1, were artificially synthesized(Genscript, Nanjing, China) and cloned into the pPICZαA expression vector from Invitrogen. This allowed for fusion expression with the alpha-mate signal peptide. The resulting plasmids—pPIC-TmxN3, pPIC-TmxN2, pPIC-TmxN1, and pPIC-TmxB—were obtained and transformed into yeast *Pichia pastoris* via electroporation. The transformants were cultured overnight in liquid YPD medium containing 20 g/L peptone, 10 g/L yeast extract, and 20 g/L glucose, and then inoculated with 1.5 mL in 25 mL of BMGY medium, consisting of 1% yeast extract, 2% peptone, and 100 mM potassium phosphate (pH 6.0). Inductive expression was carried out for 72 h, and the resulting secreted expression enzymes were purified by Ni-agarose column.

### 4.4. Kinetic Parameters Determination and Thermal Stable Characterization

The xylanase activity was measured using the 3,5-dinitro salicylic acid (DNS) method. To carry out the test, a reaction mixture was prepared by adding 300 μL of 1% wheat arabinoxylan (Megazyme Corp., Wicklow, Ireland), 100 μL of the diluted enzyme, and 100 μL of buffer with different pHs. Following a 5 min incubation period, 300 μL of DNS was added to the reaction mixture, which was then boiled for 5 min. The enzyme activity was quantified as the amount of enzyme that released 1 μmol of reducing sugars (xylose) per minute. This quantity was defined as one unit (U) of enzyme activity.

To determine the optimal temperature of the enzymes, the reaction mixture was subjected to different temperatures ranging from 30 °C to 100 °C. To check thermostability, the enzyme was incubated at 100 °C for 120 min, and the enzyme was liquated at intervals to check the remaining activity. The half-life time of an enzyme under 100 °C conditions was calculated according to the inactivation equation: ln_(Et/E0)_ = −kt, in which E0 was the relative activity at 0 timepoint, Et was the relative activity at t timepoint. The calculation formula for half-life time t_1/2_ was t_1/2_ = ln_2/k_ [40].

For assessing the pH adaptability, citric acid-Na_2_HPO_4_ buffer (pH 2.5–8.0) and glycine-NaOH buffer (pH 8.0–11.0) were used. The kinetic parameters of xylanase were investigated at 100 °C and pH 5 for 5 min, using 0.2% to 1% mg/mL of wheat arabinoxylan as the substrate. The kinetic constants were evaluated using the non-linear regression method employing GraphPad. To check the products of wheat arabinoxylan digested by xylanase, thin-layer chromatography (TLC) method was used using xylose and XOS (X2–X6) as the standard.

### 4.5. High-Density Cultivation of Recombinant Strain and Xylanase Expression in a 5L Bioreactor

The fermentation process was carried out in a 5L bioreactor, utilizing 1.5 L of basal salt medium (BSM) which was further fortified with 15 mL of PTM_1_ supplement. To begin the process, activate the yeast strain on a YPD plate and move it to a first-level seed liquid containing 4 mL of YPD medium. From there, inoculate the primary seed solution into a 1 L conical flask filled with 300 mL of YPD medium to create the secondary seed solution. Cultivate this mixture until it reaches OD20. 

Add the secondary seed solution into BSM, maintain the pH of the culture medium at 6 and the temperature at 30 °C. To ensure proper growth, increase the speed and ventilation during the initial stage of cultivation. In the phase of cellular growth, the cultivation process was carried out until the depletion of glycerol, and OD slowly increased during this period. The glycerol was exhausted about 12 h later, and a feeding medium containing 50% (*w*/*v*) glycerol along with 10 mL/L PTM_1_ solution was introduced. This was done by a pre-determined DO level of 10–20%. The glycerol feed was discontinued when OD600 reached about 100, resulting in a rise in DO levels after approximately 1 h. At this juncture, 50% methanol containing 10 mL/L PTM_1_ solution was fed to induce targeted gene expression, while the fermentation temperature was adjusted to 28 °C, with DO being set to 10%. Samples of the culture were collected every 12 h to determine OD600, the dry weight of cells, and enzyme activity.

### 4.6. Wheat Bread Making and Quality Assessment

The following recipe was used to make the bread: Golden Arowana high-gluten wheat flour (100 g), NaCl (1% *w*/*w*), yeast (1.6% *w*/*w*), water (60 mL), animal butter (4% *w*/*w*), and sugar (8% *w*/*w*), with the addition of xylanase enzymes TmxN3 (1.5, 3.0, and 4.5 mg/kg flour) and TmxB (1.5 mg/kg and 4.5 mg/kg flour). Before use, the enzyme preparations were mixed with water to ensure an even distribution throughout the dough. 

To make the bread dough, all of the ingredients were mixed together in a screw mixer for 18 min until the ideal consistency was achieved. First, it was stirred slowly for six minutes (100 rpm) without adding water. The ingredients were mixed evenly in a dry state. Then water was added and it was stirred slowly for 6 min to mix the ingredients into wet ingredients. Then it was stirred quickly for 6 min to form a dough. The mixed dough was then allowed to rest for 5 min and incubated in an incubator with 90% humidity and a temperature of 37 °C for 60 min. The weight of the dough in one mold was 100 g. The bread was then baked at 200 °C for 20 min, with three loaves made per batch. After baking, the bread was cooled to 25 °C for 2 h, sliced to a thickness of 3 cm, and quality assessment was performed.

The texture of the sliced bread samples was analyzed using a texture meter (TA. XT plus, Stable Microsystems, Shanghai, China). The sliced bread samples were compressed to 50% of their original thickness with a pre-test speed of 3 mm/s, a test speed of 1 mm/s, and a post-test speed of 5 mm/s, with a trigger force of 5 g. The interval between the two compression cycles was 5 s. Three bread samples from each baking test were used to determine the textural properties of the bread structure, including hardness, springiness, chewiness, gumminess, cohesiveness, and resilience. Statistical analysis was performed using SPSS software (Version 26.0), and analysis of variance (ANOVA) was used to compare means (*p* < 0.05) with Duncan’s multiple extreme variance tests.

## 5. Conclusions

The activity and stability of *T. maritima* xylanase were significantly improved through ancestral sequence reconstruction and molecular dynamic analysis. According to its *V_max_* value of 45,827 U/mg at 100 °C, TmxN3 is a thermophilic xylanase with high thermal stability. It has a half-life of 330 min at 100 °C, making it suitable for use in high-temperature environments. The properties of TmxN3 make it a potential additive for bread baking. When added at a dose of 4.5 mg/kg flour, TmxN3 can improve the texture of bread, particularly the hardness and chewiness. Thus, TmxN3, the new xylanase, has a positive impact on bread quality and can be used as a promising additive in bread production. The successful production of the enzyme in a 5L bioreactor has also paved the way for its future industrial use. However, its downstream purification of methanol may limit its application in the food industry, but its excellent temperature resistance may have potential applications in bio bleaching and textiles.

## Figures and Tables

**Table 1:** Interaction between substrate and active center.

	H-Bond Number	H-Bond Min-Distance	H-Bond Mean-Distance	Salt Bridge Number	Salt Bridge Min-Distance
TmxB	6	2.79	3.28	1	4.47
TmxN1	6	2.86	3.26	1	4.46
TmxN2	5	3.07	3.55	1	4.47
TmxN3	7	2.79	3.35	1	4.46
TmxSub3	5	2.48	3.17	1	4.59
TmxSub2	7	2.81	2.97	2	4.8
TmxSub1	5	2.58	3.23	2	3.85
TmxSub4	1	3.19	3.19	0	-
TmxSub5	1	3.70	3.70	1	4.93

**Table 2:** The kinetic parameters of xylanase TmxB and the ancestral partners.

Enzyme	*V*_max_ (U/mg)	*K*_m_ (mg/mL)	*K*_cat_ (s^−1^)	*K*_cat_/*K*_m_ (mL/s/mg)
TmxB	17,019	0.82	4758	5468
TmxN3	45,827	0.80	7986	9367
TmxN2	2025	0.69	3470	5028
TmxN1	1959	1.00	2163	2160

**Table 3:** The textural characteristics of wheat flour bread after storage on the shelf.

Xylanase	Hardness (g)	Chewiness (g × mm)	Gumminess	Cohesiveness	Springiness (mm)	Resilience
Control (No)	508.28 ± 39.56 ^c^	370.07 ± 70.35 ^c^	418.69 ± 71.82 ^c^	0.81 ± 0.08 ^a^	0.88 ± 0.02 ^a^	0.07 ± 0.00 ^a^
1 d	563.25 ± 42.22 ^b^	498.05 ± 24.78 ^d^	552.95 ± 51.82 ^d^	0.85 ± 0.032 ^d^	0.75 ± 0.042 ^a^	0.059 ± 0.012 ^a^
7 d	3701.48 ± 20.33 ^e^	3184.67 ± 19.80 ^f^	4421.41 ± 43.45 ^f^	0.66 ± 0.020 ^d^	0.65 ± 0.017 ^c^	0.02 ^a^
10 d	3905.47 ± 47.36 ^e^	3579.77 ± 11.75 ^e^	4729.76 ± 18.21 ^e^	0.61 ± 0.015 ^c^	0.65 ± 0.029 ^d^	0.01 ^a^
TmxB (1.5 mg/kg)	454.82 ± 38.43 ^bc^	335.82 ± 68.16 ^c^	380.04 ± 69.18 ^c^	0.83 ± 0.09 ^a^	0.88 ± 0.02 ^a^	0.08 ^a^
1 d	504.66 ± 37.58 ^b^	352.66 ± 15.71 ^c^	478.46 ± 28.43 ^c^	0.66 ± 0.0226 ^a^	0.76 ± 0.043 ^a^	0.056 ± 0.012 ^a^
7 d	2010.62 ± 37.42 ^d^	1479.08 ± 38.14 ^e^	1828.88 ± 37.24 ^e^	0.44 ± 0.025	0.45 ± 0.04 ^a^	0.02 ^a^
10 d	2682.96 ± 41.48 ^d^	3412.97 ± 23.26 ^d^	4541.45 ± 73.01 ^d^	0.43 ± 0.042 ^ab^	0.44 ± 0.01 ^b^	0.01 ^a^
TmxB (4.5 mg/kg)	401.07 ± 44.13 ^b^	296.81 ± 66.45 ^bc^	335.07 ± 66.78 ^bc^	0.82 ± 0.08 ^a^	0.88 ± 0.02 ^a^	0.08 ± 0.01 ^a^
1 d	505.46 ± 50.062 ^b^	325.06 ± 19.58 ^c^	408.76 ± 41.7 ^b^	0.76 ± 0.022 ^b^	0.76 ± 0.042 ^a^	0.06 ± 0.012 ^a^
7 d	1680.29 ± 49.59 ^c^	1316.77 ± 33.44 ^d^	1558.10 ± 43.20 ^d^	0.45 ± 0.012 ^ab^	0.47 ± 0.012 ^a^	0.02 ^a^
10 d	2444.99 ± 29.08 ^c^	3274.85 ± 27.24 ^c^	3983.09 ± 61.62 ^c^	0.44 ± 0.01 ^ab^	0.49 ± 0.0058 ^bc^	0.01 ^a^
TmxN3 (1.5 mg/kg)	290.02 ± 40.09 ^a^	214.21 ± 53.81 ^ab^	243.64 ± 51.45 ^ab^	0.83 ± 0.07 ^a^	0.87 ± 0.04 ^a^	0.078 ^a^
1 d	329.26 ± 3.23 ^a^	243.76 ± 33.202 ^b^	266.36 ± 20.42 ^a^	0.76 ± 0.022 ^ab^	0.76 ± 0.032 ^a^	329.26 ± 3.23 ^a^
7 d	1328.42 ± 47.99 ^b^	1160.25 ± 30.54 ^c^	1152.6 ± 27.24 ^c^	0.45 ± 0.023 ^ab^	0.48 ± 0.011 ^a^	1328.42 ± 47.99 ^b^
10 d	1609.21 ± 50.88 ^b^	2159.16 ± 30.13 ^b^	2391.89 ± 33.4 ^b^	0.48 ± 0.017 ^b^	0.38 ± 0.042 ^a^	1609.21 ± 50.88 ^b^
TmxN3 (3.0 mg/kg)	230.95 ± 20.01 ^a^	165.99 ± 38.75 ^a^	189.08 ± 39.51 ^a^	0.81 ± 0.11 ^a^	0.87 ± 0.03 ^a^	0.08 ± 0.01 ^a^
1 d	302.26 ± 40.21 ^a^	191.46 ± 30.48 ^a^	196.75 ± 38.41 ^a^	0.76 ± 0.042 ^ab^	0.86 ± 0.032 ^ab^	0.06 ± 0.012 ^a^
7 d	916.4845 ± 13.58 ^a^	1014.6991 ± 22.72 ^b^	1048.1843 ± 40.67 ^b^	0.51 ± 0.055 ^bc^	0.53 ± 0.025 ^b^	0.0167 ± 0.0058 ^a^
10 d	1192.6785 ± 52.21 ^a^	1927.5211 ± 25.39 ^a^	2205.6673 ± 41.09 ^a^	0.41 ± 0.017 ^a^	0.4767 ± 0.015 ^bc^	0.01 ^a^
TmxN3 (4.5 mg/kg)	228.64 ± 15.67 ^a^	177.75 ± 32.36 ^a^	194.61 ± 32.24 ^a^	0.84 ± 0.09 ^a^	0.91 ± 0.02 ^a^	0.07 ± 0.03 ^a^
1 d	276.0596 ± 16.88 ^a^	197.6596 ± 27.86 ^ab^	232.0596 ± 42.12 ^a^	0.7596 ± 0.042 ^b^	0.8596 ± 0.032 ^b^	0.057 ± 0.0025 ^a^
7 d	962.9357 ± 27.14 ^a^	911.1942 ± 35.38 ^a^	955.4933 ± 58.99 ^a^	0.5133 ± 0.031 ^c^	0.68 ± 0.036 ^c^	0.017 ± 0.0057 ^a^
10 d	1053.167 ± 14.13 ^a^	965.3332 ± 33.82 ^a^	1127.2548 ± 7.44 ^a^	0.4467 ± 0.031 ^ab^	0.5267 ± 0.042 ^bc^	0.01 ^a^

The standard deviations were calculated from three replications of each treatment. Lowercase letters indicate significant differences in values in the same column. Values with the different letters are significantly different at *p* < 0.05 according to Duncan’s multiple range tests.

**Table 4:** Application and characterization of different xylanases in bread baking.

Xylanase Family	Taxon	Optimal Temperature (°C)	Optimal pH	Enzyme Amount	Hardness Reduced	Chewiness Reduced	Reference
GH10	*Thermotoga* *maritima*	100	5	4.5 mg/kg	55.2%	40.11%	This study
GH11	*Streptomyces* sp. S27	75	6	300 U/kg	ND	ND	[30]
GH11	*Halolactibacillus miurensis*	45	6.5	6.0 mg/kg	34.73%	31.74	[31]
GH 10	*Anaeromyces robustus*	40	5.5	75 mg/kg	31.03%	34.76%	[32]
GH8	*Sorangium cellulosum*	50	6	0.2 mg/kg	50.6%	41.3%	[29]

## Data Availability

The data underlying this article will be shared on reasonable request to the corresponding author.

## References

[1] Santibáñez L., Henríquez C., Corro-Tejeda R., Bernal S., Armijo B., Salazar O. (2021). Xylooligosaccharides from lignocellulosic biomass: A comprehensive review. Carbohydr. Polym..

[2] Mendonça M., Barroca M., Collins T. (2023). Endo-1, 4-β-xylanase-containing glycoside hydrolase families: Characteristics, singularities and similarities. Biotechnol. Adv..

[3] Yang J., Han Z. (2018). Understanding the positional binding and substrate interaction of a highly thermostable GH10 xylanase from *Thermotoga maritima* by molecular docking. Biomolecules.

[4] Bajaj P., Mahajan R. (2019). Cellulase and xylanase synergism in industrial biotechnology. Appl. Microbiol. Biotechnol..

[5] Yang J., Ma T., Shang-Guan F., Han Z. (2020). Improving the catalytic activity of thermostable xylanase from *Thermotoga maritima* via mutagenesis of non-catalytic residues at glycone subsites. Enzym. Microb. Technol..

[6] Kumar V., Dangi A.K., Shukla P. (2018). Engineering thermostable microbial xylanases toward its industrial applications. Mol. Biotechnol..

[7] Golgeri M.D.B., Mulla S.I., Bagewadi Z.K., Tyagi S., Hu A., Sharma S., Bilal M., Bharagava R.N., Ferreira L.F.R., Gurumurthy D.M. (2024). A systematic review on potential microbial carbohydrases: Current and future perspectives. Crit. Rev. Food Sci. Nutr..

[8] Iqbal S., Arif S., Khurshid S., Iqbal H.M., Akbar Q., Ali T.M., Mohiuddin S. (2023). A combined use of different functional additives for improvement of wheat flour quality for bread making. J. Sci. Food Agric..

[9] Zerva A., Pentari C., Ferousi C., Nikolaivits E., Karnaouri A., Topakas E. (2021). Recent advances on key enzymatic activities for the utilisation of lignocellulosic biomass. Bioresour. Technol..

[10] Yadav P., Maharjan J., Korpole S., Prasad G.S., Sahni G., Bhattarai T., Sreerama L. (2018). Production, purification, and characterization of thermostable alkaline xylanase from *Anoxybacillus kamchatkensis* NASTPD13. Front. Bioeng. Biotechnol..

[11] Bhat S.K., Purushothaman K., Kini K.R., Gopala Rao Appu Rao A.R. (2022). Design of mutants of GH11 xylanase from Bacillus pumilus for enhanced stability by amino acid substitutions in the N-terminal region: An in silico analysis. J. Biomol. Struct. Dyn..

[12] Bello A., Giménez-Rico R.D., Gilani S., Hillen B.C., Venter K.M., Plumstead P., Dersjant-Li Y. (2023). Application of enzyme matrix values for energy and nutrients to a wheat-corn-soybean meal-based broiler diet supplemented with a novel phytase, with or without a xylanase–β-glucanase, achieved a production benefit over a nutritionally adequate unsupplemented diet. Poult. Sci..

[13] Kaushal J., Khatri M., Singh G., Arya S.K. (2021). A multifaceted enzyme conspicuous in fruit juice clarification: An elaborate review on xylanase. Int. J. Biol. Macromol..

[14] Spence M.A., Kaczmarski J.A., Saunders J.W., Jackson C.J. (2021). Ancestral sequence reconstruction for protein engineers. Curr. Opin. Struct. Biol..

[15] Chen X., Dou Z., Luo T., Sun Z., Ma H., Xu G., Ni Y. (2022). Directed reconstruction of a novel ancestral alcohol dehydrogenase featuring shifted pH-profile, enhanced thermostability and expanded substrate spectrum. Bioresour. Technol..

[16] Lei L., Zhao L., Hou Y., Yue C., Liu P., Zheng Y., Peng W., Yang J. (2023). An Inferred Ancestral CotA Laccase with Improved Expression and Kinetic Efficiency. Int. J. Mol. Sci..

[17] Zeng B., Zhou Y., Yi Z., Zhou R., Jin W., Zhang G. (2021). Highly thermostable and promiscuous β-1, 3-xylanasen designed by optimized ancestral sequence reconstruction. Bioresour. Technol..

[18] Siddiq M.A., Hochberg G.K., Thornton J.W. (2017). Evolution of protein specificity: Insights from ancestral protein reconstruction. Curr. Opin. Struct. Biol..

[19] Hochberg G.K., Liu Y., Marklund E.G., Metzger B.P., Laganowsky A., Thornton J.W. (2020). A hydrophobic ratchet entrenches molecular complexes. Nature.

[20] Singh A., Vanga S.K., Orsat V., Raghavan V. (2018). Application of molecular dynamic simulation to study food proteins: A review. Crit. Rev. Food Sci. Nutr..

[21] Scossa F., Fernie A.R. (2021). Ancestral sequence reconstruction-An underused approach to understand the evolution of gene function in plants?. Comput. Struct. Biotechnol. J..

[22] Ma M., Mu T., Sun H., Zhou L. (2022). Evaluation of texture, retrogradation enthalpy, water mobility, and anti-staling effects of enzymes and hydrocolloids in potato steamed bread. Food Chem..

[23] Kamilari E., Stanton C., Reen F.J., Ross R.P. (2023). Uncovering the biotechnological importance of *Geotrichum candidum*. Foods.

[24] Qi Y.L., Evans P.N., Li Y.X., Rao Y.Z., Qu Y.N., Tan S., Jiao J.Y., Chen Y.T., Hedlund B.P., Shu W.S. (2021). Comparative genomics reveals thermal adaptation and a high metabolic diversity in “*Candidatus* Bathyarchaeia”. Msystems.

[25] Lu Y., Fang C., Wang Q., Zhou Y., Zhang G., Ma Y. (2016). High-level expression of improved thermo-stable alkaline xylanase variant in *Pichia pastoris* through codon optimization, multiple gene insertion and high-density fermentation. Sci. Rep..

[26] Wongwisansri S., Promdonkoy P., Matetaviparee P., Roongsawang N., Eurwilaichitr L., Tanapongpipat S. (2013). High-level production of thermotolerant β-xylosidase of *Aspergillus* sp. BCC125 in *Pichia pastoris*: Characterization and its application in ethanol production. Bioresour. Technol..

[27] Liu Y., Wang J., Bao C., Dong B., Cao Y. (2021). Characterization of a novel GH10 xylanase with a carbohydrate binding module from *Aspergillus sulphureus* and its synergistic hydrolysis activity with cellulase. Int. J. Biol. Macromol..

[28] Morgan N., Bhuiyan M., Hopcroft R. (2022). Non-starch polysaccharide degradation in the gastrointestinal tract of broiler chickens fed commercial-type diets supplemented with either a single dose of xylanase, a double dose of xylanase, or a cocktail of non-starch polysaccharide-degrading enzymes. Poult. Sci..

[29] Li X., Zhang L., Jiang Z., Liu L., Wang J., Zhong L., Yang T., Zhou Q., Dong W., Zhou J. (2022). A novel cold-active GH8 xylanase from cellulolytic myxobacterium and its application in food industry. Food Chem..

[30] de Queiroz Brito Cunha C.C., Gama A.R., Cintra L.C., Bataus L.A.M., Ulhoa C.J. (2018). Improvement of bread making quality by supplementation with a recombinant xylanase produced by Pichia pastoris. PLoS ONE.

[31] Zhang Y., Liu C., Yang M., Ou Z., Lin Y., Zhao F., Han S. (2022). Characterization and application of a novel xylanase from Halolactibacillus miurensis in wholewheat bread making. Front. Bioeng. Biotechnol..

[32] Wen S., Wu G., Wu H. (2021). Biochemical characterization of a GH10 xylanase from the anaerobic rumen fungus *Anaeromyces robustus* and application in bread making. 3 Biotech.

[33] Kanehisa M., Sato Y., Morishima K. (2016). BlastKOALA and GhostKO ALA: KEGG Tools for Functional Characterization of Genome and Metagenome Sequences. J. Mol. Biol..

[34] Cameron M., Bernstein Y., Williams H.E. (2007). Clustered sequence representation for fast homology search. J. Comput. Biol..

[35] Larsson A. (2014). AliView: A fast and lightweight alignment viewer and editor for large datasets. Bioinformatics.

[36] Moshe A., Pupko T. (2019). Ancestral sequence reconstruction: Accounting for structural information by averaging over replacement matrices. Bioinformatics.

[37] Du Z., Su H., Wang W., Ye L., Wei H., Peng Z., Anishchenko I., Baker D., Yang J. (2021). The trRosetta server for fast and accurate protein structure prediction. Nat. Protoc..

[38] Morris G.M., Huey R., Lindstrom W., Sanner M.F., Belew R.K., Goodsell D.S., Olson A.J. (2009). AutoDock4 and AutoDockTools4, Automated docking with selective receptor flexibility. J. Comput. Chem..

[39] Salentin S., Schreiber S., Haupt V.J., Adasme M.F., Schroeder M. (2015). PLIP: Fully automated protein-ligand interaction profiler. Nucleic Acids Res..

[40] Gupteshwar Gupta G.G., Vikram Sahai V.S., Gupta R.K. (2014). Thermal stability and thermodynamics of xylanase from *Melanocarpus albomyces* in presence of polyols and salts. BioResources.

